# Computational Simulation of the Activation Cycle of Gα Subunit in the G Protein Cycle Using an Elastic Network Model

**DOI:** 10.1371/journal.pone.0159528

**Published:** 2016-08-02

**Authors:** Min Hyeok Kim, Young Jin Kim, Hee Ryung Kim, Tae-Joon Jeon, Jae Boong Choi, Ka Young Chung, Moon Ki Kim

**Affiliations:** 1 Korea Institute for Advanced Study, Seoul, Republic of Korea; 2 School of Mechanical Engineering, Sungkyunkwan University, Suwon, Republic of Korea; 3 School of Pharmacy, Sungkyunkwan University, Suwon, Republic of Korea; 4 Department of Biological Engineering, Inha University, Incheon, Republic of Korea; University of Lethbridge, CANADA

## Abstract

Agonist-activated G protein-coupled receptors (GPCRs) interact with GDP-bound G protein heterotrimers (Gαβγ) promoting GDP/GTP exchange, which results in dissociation of Gα from the receptor and Gβγ. The GTPase activity of Gα hydrolyzes GTP to GDP, and the GDP-bound Gα interacts with Gβγ, forming a GDP-bound G protein heterotrimer. The G protein cycle is allosterically modulated by conformational changes of the Gα subunit. Although biochemical and biophysical methods have elucidated the structure and dynamics of Gα, the precise conformational mechanisms underlying the G protein cycle are not fully understood yet. Simulation methods could help to provide additional details to gain further insight into G protein signal transduction mechanisms. In this study, using the available X-ray crystal structures of Gα, we simulated the entire G protein cycle and described not only the steric features of the Gα structure, but also conformational changes at each step. Each reference structure in the G protein cycle was modeled as an elastic network model and subjected to normal mode analysis. Our simulation data suggests that activated receptors trigger conformational changes of the Gα subunit that are thermodynamically favorable for opening of the nucleotide-binding pocket and GDP release. Furthermore, the effects of GTP binding and hydrolysis on mobility changes of the C and N termini and switch regions are elucidated. In summary, our simulation results enabled us to provide detailed descriptions of the structural and dynamic features of the G protein cycle.

## Introduction

**Fig 1:**
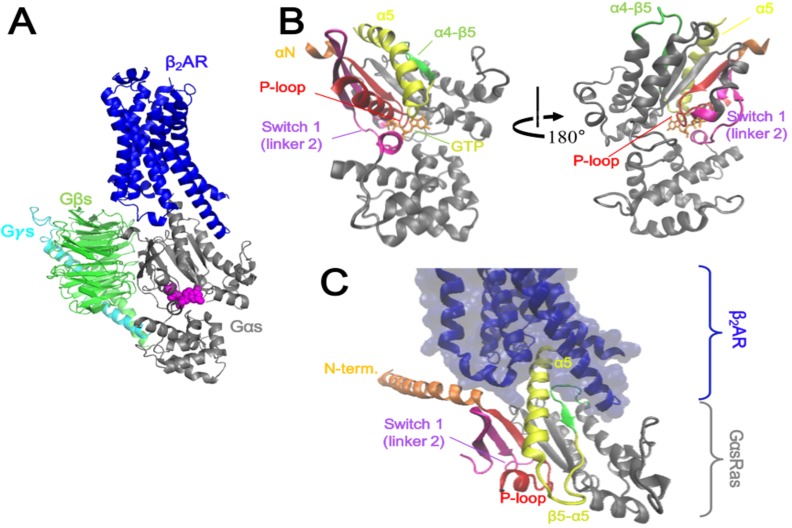
Ribbon diagrams of various components of the β_2_AR-Gs heterodimer complex. (A) β_2_AR is shown in blue, Gαs in gray, Gβs in green, Gγs in cyan and GDP in magenta. (B) Gαs structure represented by its major components for G protein activation. (C) β_2_AR-binding interface of GαsRas.

G protein-coupled receptors (GPCRs) are transmembrane receptors that have critical roles in normal physiology and pathologies of vision, olfactory perception, metabolism, the endocrine system, neuromuscular regulation, and central nervous system (CNS) [1]. G protein heterotrimers (Gαβγ) are the canonical downstream molecules of GPCRs. Agonist binding to GPCRs induces conformational changes in the transmembrane and intracellular domains, which triggers interaction with G proteins [2]. Gα subunit mediates signal transduction from activated GPCRs, and is regulated by the exchange between GDP and GTP molecules in the nucleotide-binding pocket. High resolution X-ray crystal structures of GDP-or GTP-bound G proteins have revealed that the nucleotide-binding pocket is located between the Ras and the helical domains of the Gα subunit [3–12]. Various biochemical and biophysical studies have also delineated the interface between GPCRs and G proteins; this interface includes the αN helix and the C-terminus of the Gα subunit (Fig 1)[13–16].

**Fig 2:**
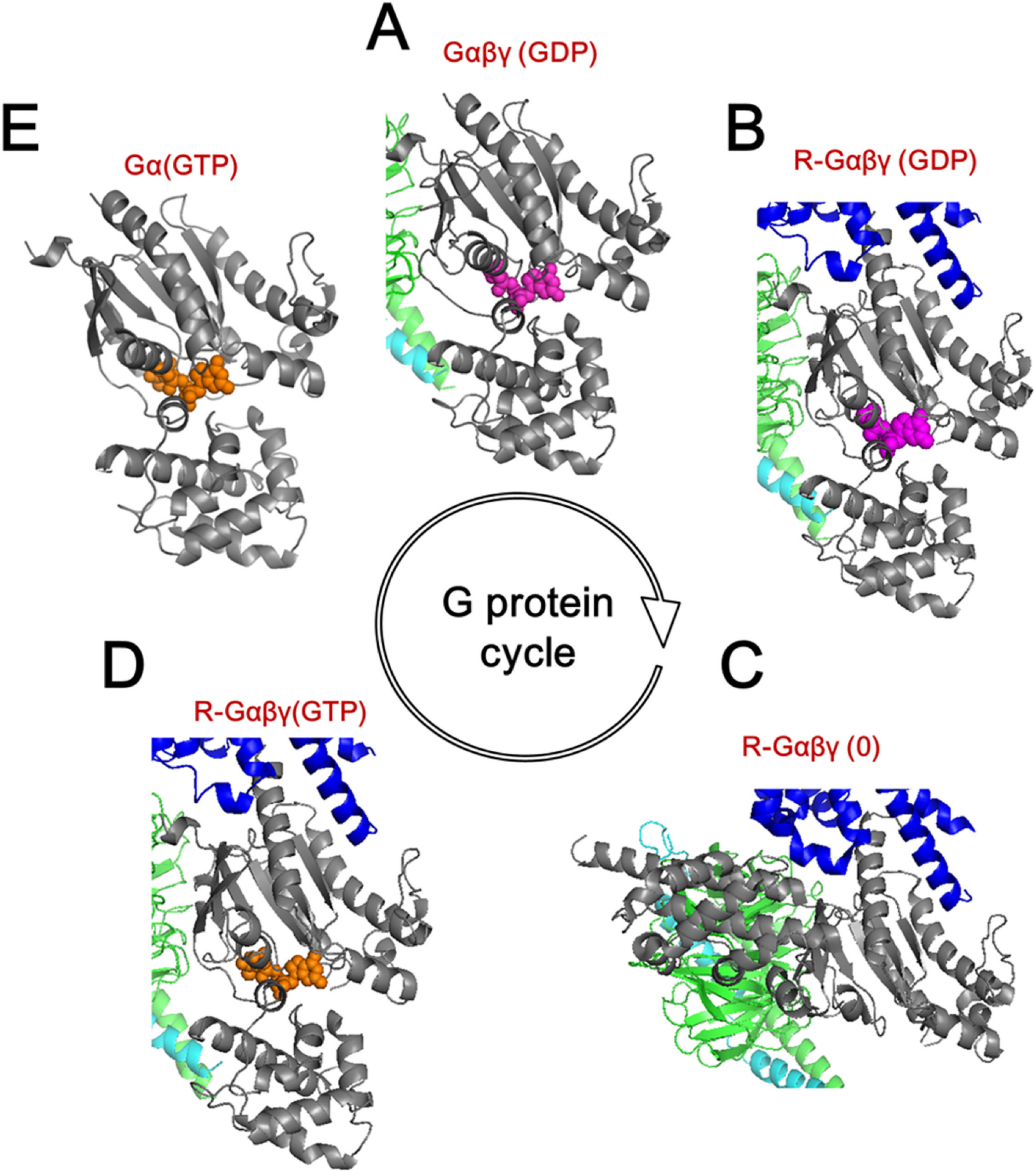
Overview of the G protein cycle. (A) Inactive GDP (*purple*)-bound Gs protein heterotrimer with α (*grey*), β (green), and γ (*cyan*) subunits. (B) Activated receptor (*blue*)-bound temporal state of Gαβγ(GDP). (C) Receptor- bound fully opened Gαβγ. (D) The receptor- bound temporal state of Gαβγ with GTP (*orange*). (E) Active GTP-bound Gα. Receptor and Gβγ subunits were truncated for convenience.

The different conformational states of the Gα subunit during its activation cycle are illustrated in Fig 2. The Gα subunit forms a heterotrimer with the Gβγ subunit when GDP is bound, while the GDP-bound Gαβγ heterotrimer is in an inactive state [17]. Agonist-activated GPCR binds to the inactive GDP-bound Gαβγ heterotrimer [Gαβγ(GDP)], which leads to the release of GDP, thereby forming a nucleotide-free GPCR/Gαβγ complex [R-Gαβγ(0)]. In the nucleotide-free conformation, the helical domain undergoes large movement and the nucleotide-binding pocket opens [18,19]. Subsequently, GTP enters the empty nucleotide-binding pocket of the Gα subunit, which results in a closed Gα conformation and simultaneous dissociation of Gβγ and GPCR [17]. Eventually, the GTP-bound Gα subunit is converted into the active form [Gα(GTP)], which activates downstream effector proteins. Because of the GTPase activity of the Ras domain, GTP is hydrolyzed to GDP, thereby transforming the structure of Gα and increasing its affinity for Gβγ; this structure forms the initial, inactive GDP-bound Gαβγ [Gαβγ(GDP)][17].

While the conformational states of the Gα subunit in the G protein cycle have been studied in detail by X-ray crystallography, electron microscopy, electron paramagnetic resonance, hydrogen-deuterium exchange mass spectrometry, and bioluminescence resonance energy transfer [18–20], the mechanisms relating signal transduction to the conformational changes of the Gα subunit between states remain elusive. For example, the mechanism of how receptors catalyze nucleotide exchanges remains to be determined [3]. A few computational studies analyzed the G protein activation process that cannot be easily addressed by experimental study [21–27]. However, these studies on the dynamic features of G protein were limited to either an individual step within the entire G protein cycle or a specific region of the Gα subunit.

In the present study, we simulated the series of structural transitions of the Gα subunit that occur during the G protein cycle. Several critical issues were addressed: how the interaction between the receptor and the G protein leads to the release of GDP; and what the effect of GTP binding to the empty receptor/G protein complex is. We used β_2_-adrenergic receptor (β_2_AR) and the Gs protein as a model GPCR and G protein pair because the X-ray crystal structure of the β_2_AR-Gs complex is the only reported high-resolution structure of the GPCR-G protein complex. We divided the G protein cycle into five conformational steps, based on two existing crystal structures (Fig 2C and 2E) [10,18] and three engineered models (Fig 2A, 2B and 2D). To describe large conformational changes during the G protein cycle, normal mode analysis (NMA) was performed based on the recently developed Mass-Weighted Chemical Elastic Network Model (MWCENM) [28,29]. Mean squared fluctuation comparison between two consecutive steps and NMA for each step clarified the role of GPCR and GTP binding in the conformational changes of Gα and the effects on each subsequent event in the G protein cycle.

## Materials and Methods

### Selection and modeling of conformational steps

Of the four types of G proteins (Gs, Gi, Gq, and G12)[30], we chose to use Gs coupled with β_2_AR as a model system. For convenience, we defined all Gs-related structures as “G” and the β_2_AR as “receptor” or “R.”

Even though only three X-ray structures of Gs are available in the Protein Data Bank (PDB) (Gα(GTPγS) [1azt.pdb], R-Gαβγ(0) [3sn6.pdb], and adenylyl cyclase-bound Gα [1azs.pdb]), we were able to model the three possible intermediate states by combining two structures (1azt.pdb and 3sn6.pdb) from other classes of G proteins (Gt and Gi).

The major geometrical differences between the inactive Gαβγ(GDP) model and the Gα(GTPγS) model are the type of nucleotides bound (GDP/GTP) and the presence of Gβγ. Because the structure of Gi has been defined for both forms, inactive and active [13], we adopted inactive and active Gi structure to model the structure of GDP-bound Gαβγ heterotrimer. By applying these topological differences, we established an engineered Gαsβγ(GDP) model (Fig 2A). The sequence of Gαs was maintained; only the topological information from Gαi was adopted. This engineered structure was refined by the energy minimization package in Swiss-PDB Viewer [31]. A detailed description of how the engineered Gαsβγ(GDP) model was made is provided in supplementary information (See S1 Fig).

For the engineered R-Gαβγ(GDP) model, the Gα model was constructed by combining the GαsRas domain from R-Gαβγ(0) (PDB ID: 3SN6) and the GαsAH domain from Gαβγ(GDP). We made two assumptions. First, when the receptor is bound to Gα, the GαsRas domain undergoes a conformational change by forming an interface with the receptor while the GαsAH domain maintains its structure. Second, during the subsequent opening motion of Gα, GαsRas becomes firmly attached to the activated β_2_AR and has no degree of mobility [19]. Moreover, GαsAH, except in the linker 2 region, also undergoes a rigid body movement. Therefore, the intermediate R-Gαβγ(GDP) model was generated based on the Gαβγ(GDP) model with the replacement of GαsRas by the corresponding region of R-Gαβγ(0) (See S2 Fig). This engineered structure was stabilized by energy minimization as described above.

The R-Gαβγ(GTP) model (Fig 2D) is an energetically unfavorable structure but is a plausible intermediate state between R-Gαsβγ(0) (Fig 2C) and Gα(GTP) (Fig 2E). The basic concept for rendering this model is the same as described for the previous case, i.e., the two sub-domains of Gα show rigid body motion with uniform conformation during the closing motion of Gα. Therefore, this engineered complex, R-Gαβγ(GTP), is very similar to R-Gαβγ(GDP) except for the type of nucleotides. Energy minimization is subsequently conducted to obtain the refined structure.

### MWCENM with NMA

As an elastic network models (ENM), MWCENM has several advantages with regard to simulation accuracy and computational efficiency because not only the elastic network optimized with various stiffness values according to the types of chemical interaction as listed in S1 Table, but the inertial effect of each amino acid as a concentrated mass at the representative α-carbon is taken into account [28]. S3 Fig compares traditional distance-cutoff based ENM with MWCENM. In our simulation model, nucleotides in Gα model (GDP/GTP) were coarse-grained with mass-weighed representative atoms tightly connected to each other by covalent bonds, and to surrounding Gα atoms with hydrogen bonds and van der Waals (vdW) forces (see S4 Fig). Moreover, all the constraints from the receptor and Gβγ subunit are applied to the Gα model in the same manner as listed in S1 Table, which lower the computation burden as same level of single Gα model. This method can generate more plausible conformational changes than traditional ENMs due to use of a realistic connection matrix, especially in the closed form of proteins such as Gα in the B structure model of Gα protein. Moreover, higher b-factor correlation and its invariance to cutoff distance (see S5 Fig) supports our choice of MWCENM as our simulation model [28]. In MWCENM, the total kinetic energy in a network of *n* point masses is given by

$$T=\frac{1}{2}\sum_{i=1}^{n}m_{i}{\|{\dot{x}}_{i}(t)\|}^{2}$$ (1)

where *m*_*i*_ corresponds to a specific concentrated mass value according to the amino acid type, *x*_*i*_ (*t*) is the position of the *i*th atom at time *t*, and dot represents a time derivative. In addition, the total potential energy is given by:

$$V=\frac{1}{2}\sum_{i=1}^{n-1}\sum_{j=i+1}^{n}k_{i,j}{\left\{\left\|x_{i}(t)-x_{j}(t)\right\|-\left\|x_{i}(0)-x_{j}(0)\right\|\right\}}^{2}$$ (2)

where *k*_*i*,*j*_ is a spring constant between the *i*th and *j*th atom based on S1 Table. After the construction of MWCENM, we used NMA to study the dynamics of the target proteins. In NMA, the equation of motion is derived from Lagrangian mechanics as follows:

$$\frac{d}{dt}(\frac{\partial L}{\partial {\dot{\delta }}_{i}})-\frac{\partial L}{\partial \delta }=0$$ (3)

where *L* = *T* - *V* and *δ*_*i*_ is the *i*th component of the generalized deviation vector $\delta \in R^{3N}$. This physically represents a small fluctuation from the initial position of the *i*th atom *x*_*i*_ (0) so that *x*_*i*_(*t*) = *x*_*i*_(0) + *δ*_*i*_(*t*). From Eqs 1 to 3, we can derive the following equation of motion (Its full derivation is available at Ref. [32]):

$$M\ddot{\delta }+K\delta =0$$ (4)

where *M* is the mass matrix (diagonal matrix) and *K* is the stiffness (Hessian) matrix defined by *K*_*i*,*j*_ = ∂^2^*V*/∂*x*_*i*_∂*x*_*j*_. From this equation of motion, one can calculate eigenvectors and eigenvalues by diagonalization of the mass-weighted Hessian matrix, *M*^−1/2^*KM*^−1/2^, for a given protein system, which describe the vibrational frequencies and corresponding vibration modes, respectively [33].

### Elastic Network Interpolation

We used the ENI method to generate the anharmonic pathways for conformational transitions between two metastable conformations (closed R-Gαβγ(GDP) and open R-Gαβγ(0))[34,35]. In this study, we generated intermediate conformations by solving the following potential-like cost function, in which the initial conformation (closed R-Gαβγ(GDP)) is deformed toward the target conformation (open R-Gαβγ(0)) to minimize this function:

$$C(\delta )=\frac{1}{2}\sum_{i=1}^{n-1}\sum_{j=i+1}^{n}\gamma_{i,j}{\left\{\left\|x_{i}+\delta_{i}-x_{j}-\delta_{j}\right\|-l_{i,j}\right\}}^{2}$$ (5)

Here, *l*_*i*,*j*_ is the ideal length at a certain intermediate state determined by distance interpolation. *l*_*i*,*j*_ can be expressed as

$$l_{i,j}=(1-\alpha )\left\|x_{i}-x_{j}\right\|+\alpha \left\|\chi_{i}-\chi_{j}\right\|$$ (6)

where *α* is the coefficient that specifies the extent to which a given state is along the pathway from {*x*_*i*_} towards the target conformation {*χ*_*i*_}. The linking matrix *γ* is similar to that of MWCENM, but different because it is defined as a union matrix between two linking matrices for {*x*_*i*_} and {*χ*_*i*_} in the sense that *γ*_*i*,*j*_ has a value of 1 when residues *i* and *j* are within the cutoff range of 12 Å in either conformation. For more details, refer to reference [32]. Energetically, intermediate states show the higher energy values than those of the given two conformations. The pathway generated from ENI was validated using overlap values that were obtained by comparison with the results from NMA.

Matlab codes for both MWCENM and ENI are available for download on the online morph server KOSMOS (http://bioengineering.skku.ac.kr/kosmos)[36].

### Overlap Value

The overlap value is widely used as a measure of similarity between the direction of conformational change and the direction given by normal mode. The general overlap value [37] is defined as

$$O_{j}=\frac{\left|\sum_{i=1}^{3N}a_{i,j}\Delta r_{i}\right|}{{\left|\sum_{i=1}^{3N}a_{i.j}^{2}\sum_{i=1}^{3N}\Delta r_{i}^{2}\right|}^{1/2}}$$ (7)

where *O*_*j*_ is the overlap value between the conformational change vector and the *j*^th^ normal mode vector. *a*_*i*,*j*_ is the *j*^th^ eigenvector of the *i*^th^ representative atom, and *r*_*i*_ is the displacement vector of the *i*^th^ atom after two given structures are superimposed. A higher overlap value indicates higher similarity and modeling accuracy. An overlap value of 1 indicates that the computed normal mode vectors are perfectly matched to the direction of conformational change. However, in a large deformation such as the global hinge motion of Gα, the conformational change vector is not comparable with the normal mode vector because it does not represent the instantaneous direction any more [38]. Alternatively, one can compare it with normal mode vectors obtained from each intermediate conformation between two endpoint structures.

## Results and Discussion

### 1. Conformational change of Gαs upon β_2_AR binding (A➔B➔C in Fig 2)

#### 1–1. Possible binding sequence of Gαs to β_2_AR

When the Gs subunit interacts with activated β_2_AR, several parts of Gαs undergo structural changes in an energetically favorable direction. In particular, the αN helix (the N-terminus) and the C-terminus of Gα are well-mapped GPCR-binding sites that act as two conduits between the interface of the activated GPCR and the nucleotide-binding pocket [16,39–42]. Previous studies also highlighted these two regions as the key determinants of conformational changes in Gα when a GPCR-binding event occurs [18,43]. However, the binding sequence is still under debate. Herrmann and his coworkers suggested that rhodopsin first interacts with lipid moieties on the N-terminus of transducin and subsequently with the αN/β1 hinge of Gα, thereby making the C-terminus of Gα available for rhodopsin binding [44,45]. However, sequential interactions may differ according to the type of G protein and the experimental systems.

**Fig 3:**
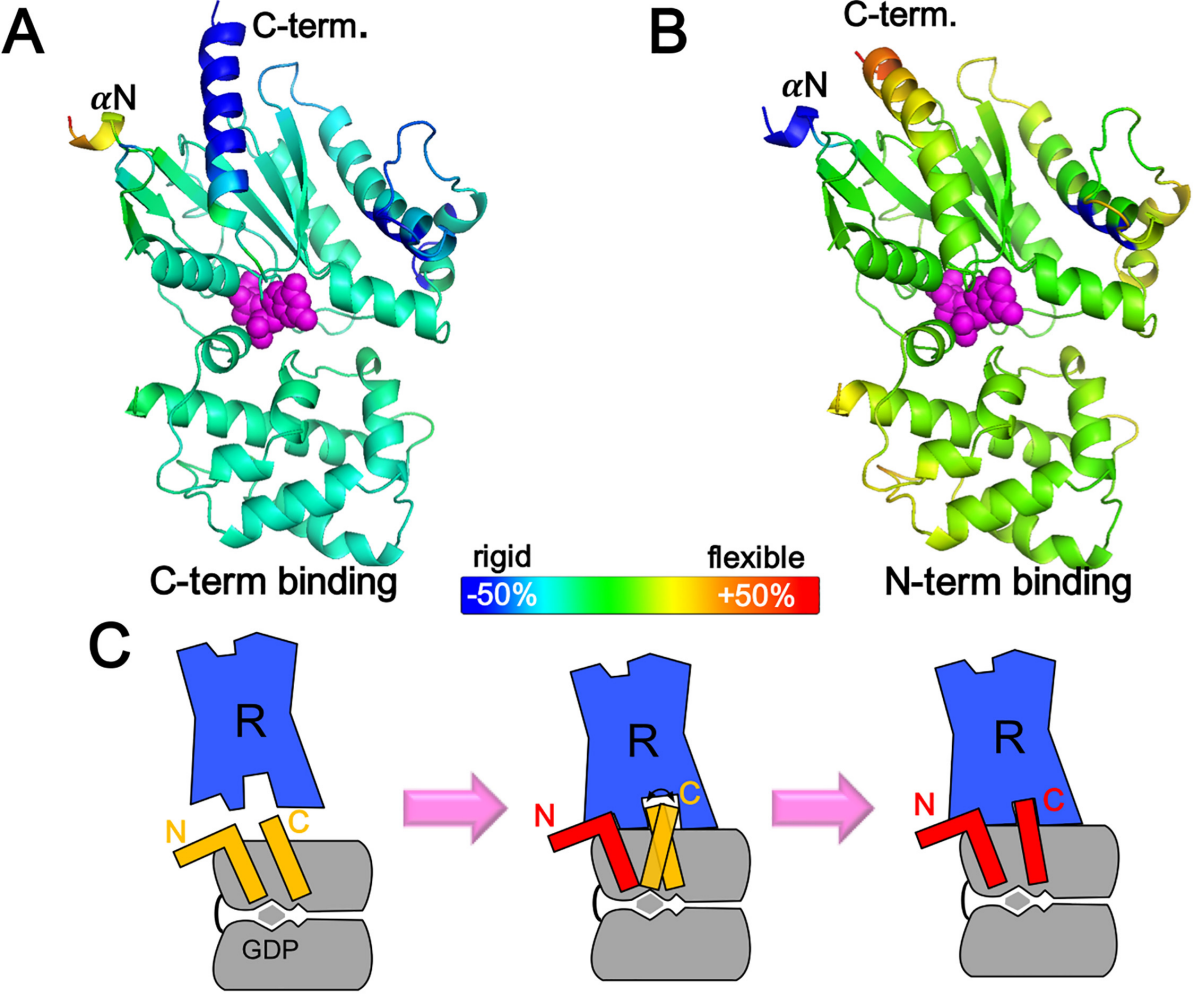
**Mobility change of Gαs after the initial binding to β**_**2**_**AR either at the C-terminus (A) or the N-terminus (B).** The theoretical value at each residue was calculated by mean squared fluctuation between Gαβγ(GDP) and pre-R-Gαβγ(GDP) in percentile and is displayed according to the indicated color map. The residue in blue (red) is considered to become rigid (flexible). (C) Schematic of the proposed binding event between Gαs (gray) and β_2_AR (blue). Activated β_2_AR may initially be bound to the N terminus of Gα, leading to a relatively flexible C terminus, which increases the probability that it will find its proper binding position to the receptor. Eventually, this binding event is completed by the C terminal binding.

In this study, we conjectured the binding sequence of the initial interaction between Gαs and β_2_AR based on the change in mean squared fluctuation. As shown in Fig 3, we assumed two possible pre-R-Gαβγ(GDP) structures, one in which the N-terminus was bound to the activated GPCR while the C-terminus remained unbound, and vice versa. It should be noted that the majority of αN is missing from existing high-resolution structures of nucleotide-bound Gs proteins. However, we conducted our analysis as described below because the β_2_AR binding interface at the N-terminus is the αN/β1 hinge, which is defined in the high-resolution structures. Each pre-R-Gαβγ(GDP) was constructed based on the Gαβγ(GDP) structure with replacement of one terminus by the corresponding part from the modeled R-Gαβγ(GDP) structure (the modeling process is described in the Materials and Methods). Only the replaced terminus was assumed to be connected to the receptor. Our basic premise is that if the mean squared fluctuation value of the target region increases, a binding event is very likely to occur in that region. Under the condition that one part of a protein is already bound to the target, higher mobility of the other part would better facilitate completion of the next binding event to the fixed target. In the conformational selection model, which is one of the hypotheses to explain protein-protein docking mechanisms like an induced fit, a flexible structure has a high likelihood of accessing target conformations due to wide ensemble of conformations [46]. It also makes sense in that a highly flexible structure usually has a low energy barrier in an energy landscape, which facilitates conformational changes [47].

In both cases, binding to the first terminus leads to high mobility of the other terminus, which indicates that once either terminus binds to the receptor, sequential binding of the other terminus occurs more easily. Even though this does not clearly indicate the most favorable binding sequence, we assume that initial binding involves the N-terminus, because initial binding at the N-terminus leads to more flexibility in the C-terminus than vice versa (Fig 3).

The average decrements in mean squared fluctuation upon initial binding of the C-terminus and N-terminus was 12.9% and 7.6%, respectively. Even though the initial bindings of either terminus decreased the flexibility of the overall structure, the decrease in mean squared fluctuation was much higher for initial C-terminus binding. This result implies that initial binding of C-terminus would impose more constraints on the mobility of GαsRas, so that it would be much harder to bind to the receptor or to release GDP compared to the case of initial N-terminus binding. Based on this, we hypothesize that the N-terminus interacts first with the receptor and then the C-terminus binds. This proposed binding event is summarized in Fig 3.

#### 1–2. Structural change of Gαs upon receptor binding–GDP release mechanism 1

**Fig 4:**
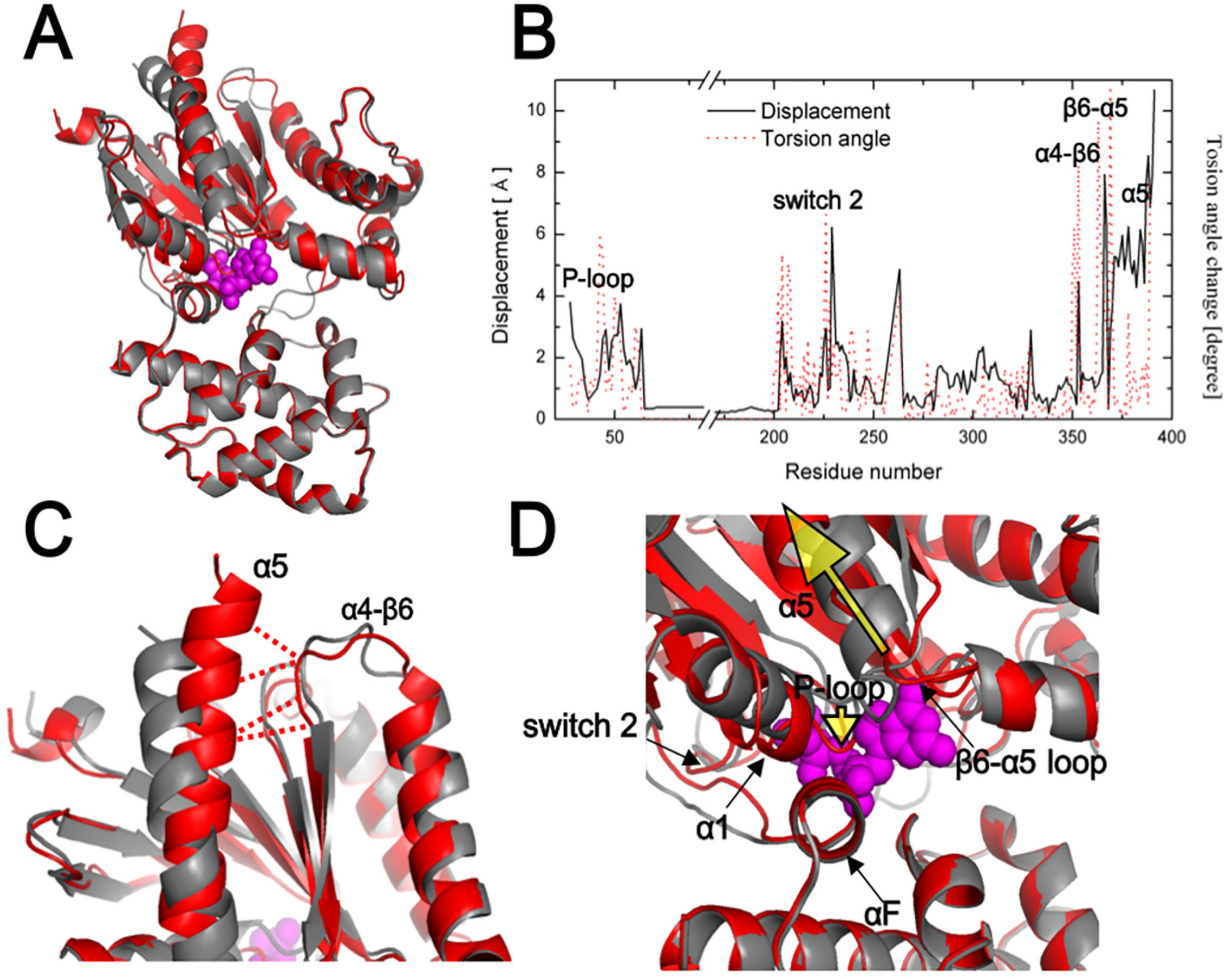
Topological changes of the Gαs protein upon β_2_AR binding. The Gαs structures in the Gαβγ(GDP) and R-Gαβγ(GDP) states are colored in gray and red, respectively. (A) Comparison of the structures of Gα with and without β_2_AR. Compared to the GαsAH domain, the GαsRas domain shows marked displacement. (B) Topological change (displacement and torsional angle) of Gαs upon β_2_AR binding. (C) The receptor-binding interface of Gαs. The distance between the α5 helix and α4-β6 decreases to 7 Å, which is within the interaction range. Red dotted lines represent new interactions between α5 helix and α4-β6 (D) Nucleotide-binding pocket (switch 2, P-loop, β6-α5 loop, and α5 helix). A significant change in the topology occurs near the nucleotide-binding pocket. The directions of movement of the α5 helix and P-loop are indicated by the yellow arrows.

It is essential to understand the structural changes of Gα upon coupling to the receptor. In fact, the region with the highest structural change is considered the greatest contributor to mutual binding effects. Direct structural comparison of Gαβγ(GDP) and R-Gαβγ(GDP) revealed specific regions known to be important for Gαs and β_2_AR coupling. As described above, Gαs underwent a dramatic conformational change at the C and N-termini of GαsRas, the interfaces for the interaction with β_2_AR (Fig 4A and 4B). Moreover, the α5 helix, which undergoes the most remarkable change [48], rotated and translated into the proximity of the α4-β6 under the uniform intrinsic conformation shown in a previous study (Fig 4B and 4C)[2]. As a result, it formed a new strong interaction with the α4-β6 loop (Fig 4C, red dotted lines).

Other perturbed regions, including the GαsAH domain, are quantitatively defined in Fig 4B. Interestingly, the nucleotide-binding pocket consisting of β6-α5, the P-loop, and switch 2 also showed notable structural changes related to the release of GDP (Fig 4B and 4D)[49]. Because of the distortion within the β6-α5 loop induced by the upward movement of the α5 helix away from the GDP molecule, the P-loop moved very close to GDP, i.e., acted like a push button in the range of repulsive vdW forces. Another possible scenario of GDP release in this pocket region is that a concerted motion is triggered by the P-loop movement and then the subsequent distortion of the α1 helix leads to disconnection with β6-α5 and GDP [50]. It is likely that these concerted motions may be the first step of GDP release from the nucleotide-binding pocket. These hypotheses provide some support to a previous study in which receptor binding was shown to disrupt the interaction between β6-α5, the αF helix, and the αA helix [19].

#### 1–3. Dynamic features of Gαs at R-Gαβγ(0)–GDP release mechanism 2

**Fig 5:**
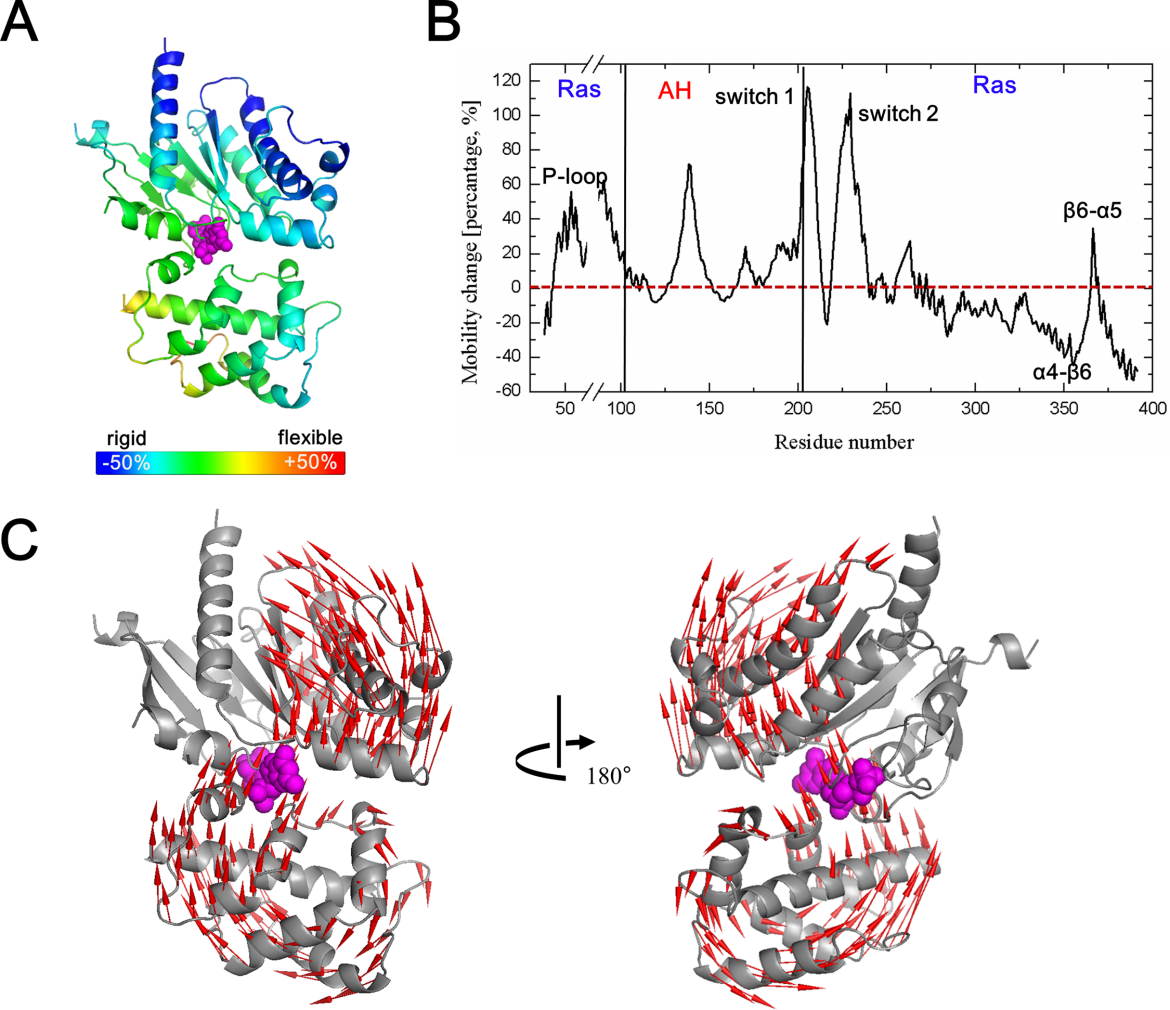
Dynamic features of the R-Gαβγ(GDP) complex. (A and B) Change in the mean squared fluctuation of Gαs upon β_2_AR binding. Mobility changes of switches 1 and 2, P-loop, β6-α5 loop, α4-β6 loop, and α5 helix are marked here. Overall, the GαsRas domain loses its intrinsic flexibility due to direct binding to the receptor. (C) Shape of R-Gαβγ(GDP) in the fourth normal mode. The open motion corresponding to the conformational change direction in Gα is shown.

The abovementioned β_2_AR coupling to Gs caused geometrical changes and perturbation of relevant connection states. On the basis of this force field difference and according to the topological changes, we analyzed the dynamic features of Gαs coupled to β_2_AR to determine the sequential GDP releasing mechanism. Comparison of the mean squared fluctuation values of Gαβγ(GDP) and R-Gαβγ(GDP) clearly demonstrated mobility changes at the atomic level (Fig 5). A mobility change was defined as the difference between two mean squared fluctuation values in percentile. It is not surprising that the major receptor binding regions, which include the C-terminus, αN, and α4-β6, showed decreased mean squared fluctuation values due to newly introduced constraints upon receptor interaction (Fig 5A and 5B)[2,10,51,52]. The most constrained regions with the lowest flexibility would be the most sustainable for the sequential reaction and interactions among all residues in Gα. These results are in good agreement with a previous study where structural mass spectrometry was employed to demonstrate that the C-terminus is immobilized by interaction with activated receptors [53]. In contrast, highly mobile regions were concentrated at the nucleotide-binding pocket and surrounding regions (Fig 5A and 5B). Not only the remarkable structural changes, as described in the previous section, but also the high mobility of the P-loop, β6-α5 loop, and switch 2 demonstrates that β_2_AR binding ultimately leads to the release of GDP through perturbation of a specific region in the nucleotide-binding pocket [54].

Another interesting region with a high mean squared fluctuation value was the switch 1 (linker 2) region (Fig 5A and 5B), which connects two Gα subdomains. The high mobility of the linker in Gα provides a basis for the sequential opening motion [55–57], which is another mechanism for receptor-mediated release of GDP. NMA of R-Gαβγ(GDP) yielded a precise opening motion of Gα in the fourth mode (Fig 5C). In this dominant mode, as expected, the GαsAH and GαsRas domains had moved in opposite directions to each other with a hinge point in the linker region, which is favorable for the release of GDP. In addition, the exit route caused by this opening motion showed good agreement with previous studies that reported that GDP release occurs on the phosphate-binding side [49,58]. In the case of Gαβγ(GDP), bending and twist modes with unchanged nucleotide-binding pocket dominantly occur at fourth and fifth modes. Because of the very tight binding between the sub-domains, the nucleotide-binding pocket was completely blocked (“like a pearl in the shell”), making it difficult for GDP to exit. Similar results were observed in the Gα structure without GDP (see S6 Fig).

An open question is whether the dissociation of subdomains is the cause or effect of GDP release [59]. Because the solvent accessible area at the nucleotide pocket of R-Gαβγ(GDP) was slightly increased in dominant opening mode (see S2 Table), in contrast to the twist and bending modes of Gαβγ(GDP) and Gαβγ(0), the opening mode was topologically more favorable than the other modes for the release of GDP. In addition, normal mode results of the R-Gαβγ(0) complex were very similar to those of R-Gαβγ(GDP) (see S6 Fig). This strongly suggests that the necessary condition for the opening motion of Gα is not the absence of GDP, but the structural change caused by β_2_AR binding. Therefore, the release of GDP may not trigger the dissociation of Gα but it just requires the receptor binding first to increase the GDP solvent-accessible surface area.

A recent simulation study also suggested that domain opening occurs spontaneously even in the absence of GDP and that domain opening is necessary but not sufficient for rapid GDP release because the receptor-induced structural rearrangement in the Ras domain of Gα is also needed for GDP release [60]. Another study reported that water molecules could enter the nucleotide-binding pocket and interact with GDP or surrounding residues during opening of the nucleotide-binding pocket [49]. In conclusion, multiple mechanisms are likely to contribute to receptor-induced GDP release. Structural and dynamical changes in the nucleotide-binding pocket may cooperate with the flexible linker region, which would weaken the interaction between Gα and GDP and also expose the bound GDP to the solvent.

### 2. A feasible pathway for Gα conformational transition (B➔C in Fig 2)

As reported previously, R-Gαβγ(0) shows a large conformational change of about 130° rotation with respect to R-Gαβγ(GDP)[58]. This observation contributed to our decision to study the conformational transitions between the two states to understand the underlying mechanism. We defined the states of R-Gαβγ(GDP) and R-Gαβγ(0) as closed and open forms of Gα, respectively, and generated a series of intermediate conformations using elastic network interpolation (ENI) (see Materials and Methods section).

**Fig 6:**
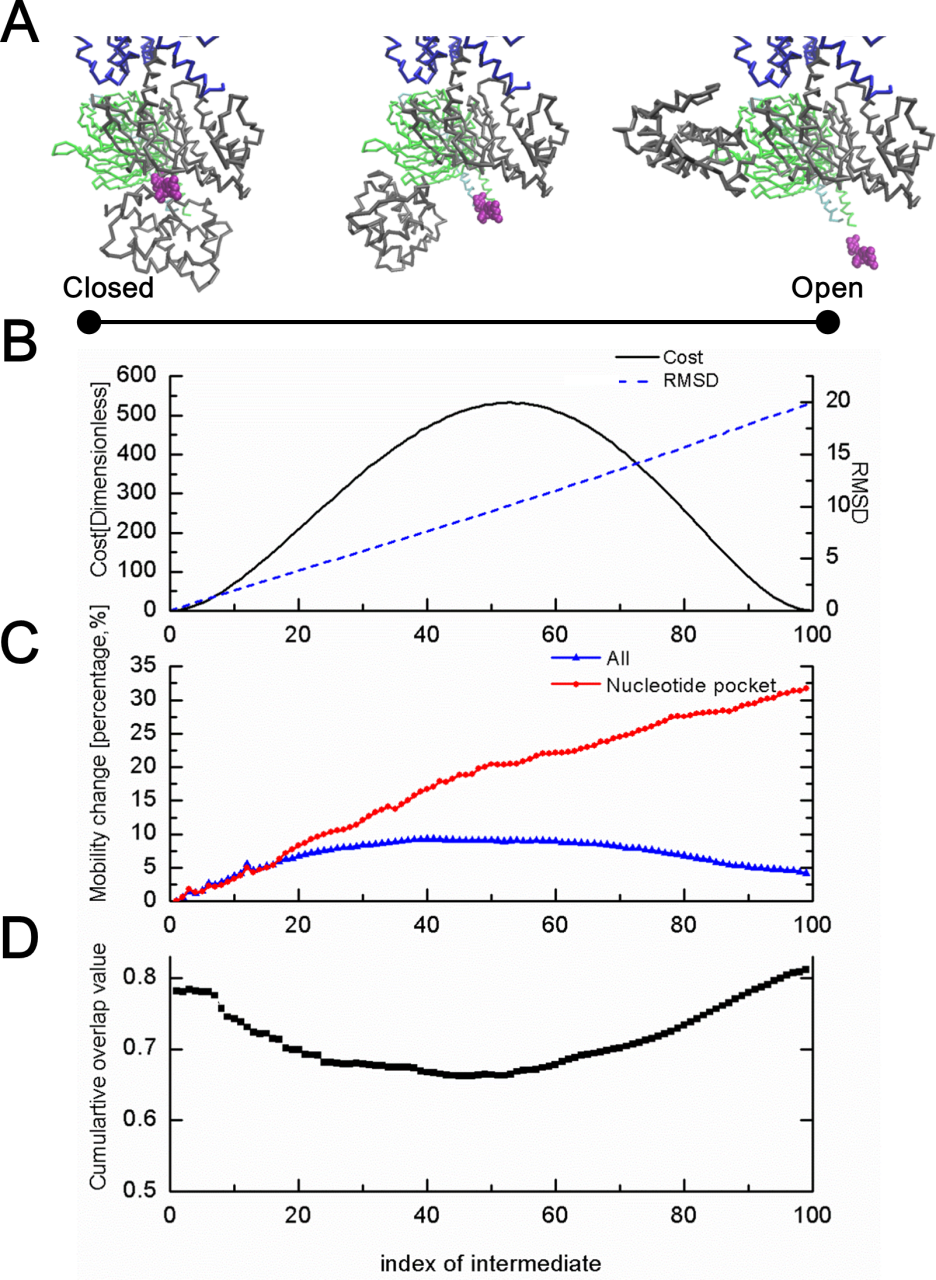
Simulation results of intermediate conformation states between the closed and open Gαs structures obtained using elastic network interpolation (ENI). (A) Possible states of Gαs. A large conformational change was observed around the GαsAH domain showing the swing motion with respect to the fixed GαsRas domain. (B) Root-mean-square deviation and cost function values of all intermediate conformational states with respect to the initial, closed Gαs structure. The smooth changes in both values represent the conformational changes and fluctuations in energy between the two reference structures of Gαs well. (C) Mobility changes during the transition. Intermediate conformations were more flexible than the two endpoint structures. Particularly, the mobility of the nucleotide-binding pocket region kept increasing during the transition. (D) Verification of the ENI pathway of the Gαs structure using the cumulative overlap values between the set of first 5% lowest normal modes and the directional vector to the target structure from each intermediate.

Simulation results for the conformational transition from the closed to the open Gα structure are shown in Fig 6A. Despite the large conformational change of Gα, the ENI method reliably generated a pathway of feasible and continuous intermediates showing smooth changes in root-mean-square deviation and cost function values (Fig 6B). Fig 6C shows the mobility changes of each intermediate conformation relative to the closed Gα conformation. The intermediate conformations always show higher mobility and the highest one is observed near the 40th intermediate conformation, which is a quantitative indication of being more flexible and excited intermediates to overcome the ground state barrier composed of the two end conformations. In particular, the nucleotide-binding pocket became very flexible (approximately 33% increase in mobility), compared to the overall intermediate structure, providing space for easy release of GDP (Fig 6C). In addition, the high overlap values between normal modes and the directional vector to the target conformation from each intermediate conformation gave quantitative support to our simulation results. In this comparison, the first 5% lowest normal modes of each intermediate were used to compute the cumulative square overlap (CSO). Fig 6D shows that the overlap value, which quantifies the ability of the proposed transition pathway to follow the target direction to the R-Gαβγ(0) conformation, was higher than 0.65 over the entire pathway.

In addition, the virtual torsion angle difference between R-Gαβγ(GDP) and R-Gαβγ(0) was calculated (S7 Fig). Significant angle changes were observed between residues 58 and 88 and around residue 200, both of which are known as linker regions that connect the GαsAH and GαsRas domains (yellow dotted and ribbon in S7 Fig respectively). In contrast, the other parts of the Gαs, including all secondary structures, changed only slightly. These results indicate that the flexible linker regions induced the large conformational changes of Gαs without any intra-conformational change in the two subdomains.

### 3. Conformational change of Gαs upon GTP binding (C➔D➔E in Fig 2)

**Fig 7:**
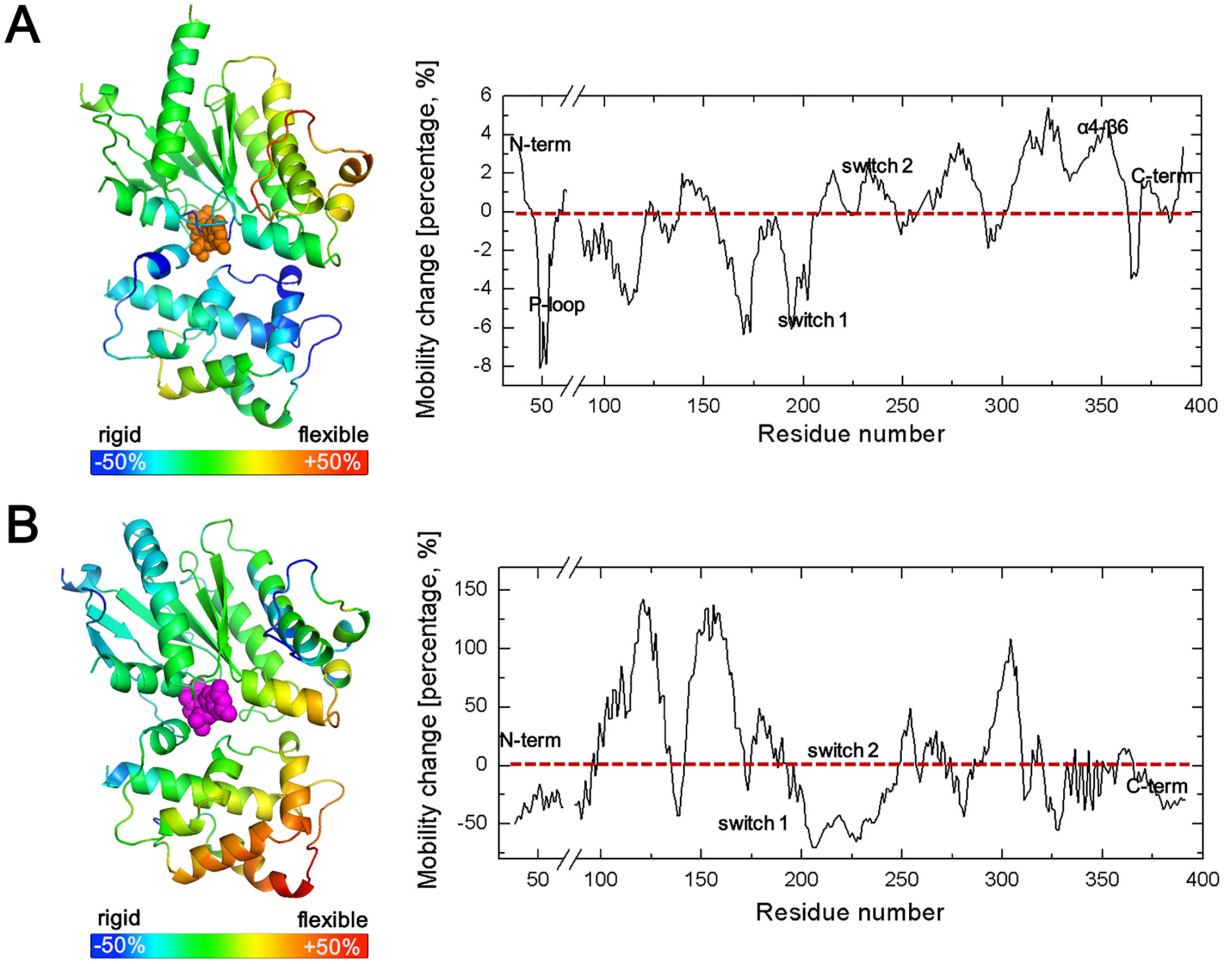
Role of GTP and its hydrolysis in Gαs mobility. (A) Change in mean squared fluctuation of Gαs in R-Gαβγ(0) following the addition of GTP. Receptor binding regions (N terminus, C terminus, α4-β6 loop) and Gβγ binding regions (switch 2) became highly mobile for easy dissociation of bound structures. In contrast, the mean squared fluctuation values of nucleotide-binding pocket regions (P-loop, switch 1) decreased, thereby blocking the opening motion between the two sub-domains of Gαs. (B) Change in mean squared fluctuation of Gαs following formation of Gαβγ(GDP) relative to Gαs in Gα(GTP). The change in mobility reflects the effects that GTP hydrolysis and Gβγ binding have on Gα.

It is apparent that GTP allows the G protein cycle to continue by dissociating the receptor and Gβγ from Gα; however, the GTP-induced conformational changes in Gα are not yet clearly understood. In this section, we focus on the changes in the mobility of Gα upon GTP binding. Comparison of the calculated mean squared fluctuation values between R-Gαβγ(0) and R-Gαβγ(GTP) (Fig 7A) specifically demonstrated the changes in mobility of Gα, which can explain the mechanism of dissociation of the receptor and Gβγ. First, major regions of the GPCR-G protein interface, such as αN, the C terminus, and the α4-β6 loop were disordered to enable decoupling of β_2_AR. Second, not large but relatively significant increases in mean squared fluctuation values in the switch 2 region contributed to the high mobility required for the dissociation of Gβγ, which resembles the environment of residues in switch 2 illuminated by site-specific fluorescence [61]. Interestingly, the mean squared fluctuation values decreased in two major regions, the P loop and switch 1 (linker 2) corresponding to the nucleotide-binding pocket and the critical connection loop for the two Gα subdomains, respectively. To retain the closed form of Gα, these regions need to be stabilized by binding of GTP [54]. In conclusion, mobility changes of Gα caused by GTP binding enabled subsequent conformational changes to the closed form of Gα resulting in dissociation of the receptor and Gβγ.

### 4. GTP hydrolysis and the effect of Gβγ binding on Gαs (E➔A in Fig 2)

After dissociation of Gβγ and β_2_AR from Gαs(GTP), the fully active GTP-bound Gα can interact with downstream effector proteins. Activation is terminated by the hydrolysis of GTP to GDP through the intrinsic GTPase activity of Gα. Moreover, the following Gα(GDP) structure is known to show a conformational change which subsequently facilitates the binding of Gβγ. Structural comparison between Gαβγ heterotrimer [4,6] and Gα(GTPγs) [9,12] revealed that the Gβγ binding region in Gα forms a different structure from GDP-bound state to GTP-bound state; with GTP, the Gβγ binding region in Gα alters so that Gβγ cannot interact with Gα. To determine the effects of GTP hydrolysis and sequential Gβγ binding to Gα, the mean squared fluctuation values of Gα(GTP) and Gαβγ(GDP) were compared. As expected, mobility (Fig 7B) decreased in switches 1 and 2 because of the generation of an additional constraint with Gβγ. Interestingly, the mobility of the C and N termini decreased, which contributed to sequential β_2_AR binding. Suppose that two uncoupled proteins randomly vibrate in solution. In this case, less fluctuation (i.e., low mobility) would be much more favorable for protein-protein interaction. As a result, GTP hydrolysis and Gβγ binding both play important roles in Gα(GDP) stabilization, which is required for the next signaling cycle.

## Conclusions

We determined various static conformations of Gα during the G protein signaling cycle based on analysis of X-ray crystallography data. In particular, the recently published crystal structure of an active state ternary complex, composed of agonist-occupied monomeric β_2_AR and nucleotide-free Gs heterotrimer, contributed to a better understanding of the Gα cycle [18]. In our simulation, based on the elastic network model (ENM), we analyzed these critical intermediate structures and connected them with dynamic processes. To gain insight into the role of protein and nucleotide binding in the conformational changes of Gα, we hypothesized the existence of the intermediate complexes of R-Gαβγ(GDP) and R-Gαβγ(GTP), which are virtual but plausible states in the G protein cycle. These intermediate structures might exist in nature, but they have not yet been reported [62].

Comparison of the mobility and structure of Gαβγ(GDP) and R-Gαβγ(GDP) showed that both N and C termini of Gα act as binding sites for β_2_AR and conformational changes in the nucleotide-binding pocket of Gα induce the release of GDP. Furthermore, our data indicated that, in terms of mutual dynamic mobility, the GPCR-G protein binding event preferentially occurs at the N terminus first, and then the C terminus. Topological changes, including perturbation in the nucleotide-binding pocket, provide a mechanism for how the receptor alters Gα to release GDP. Concerted motions of distortion within the β6-α5 loop and P loop movement are the first steps for opening the tightly closed pocket to allow nucleotide exchange.

Another interesting observation is collective motion between GαsAH and GαsRas. The opening motion at the fourth mode shape of R-Gαβγ(GDP) indicated that the substantial decrease in the linking numbers around the nucleotide-binding pocket results in a large conformational change and also determined its opening direction at the phosphate-binding side, which was proposed in a recent study using targeted molecular dynamics [58]. The high overlap values between the NM and ENI results of Gα validate our simulation results. However, our finding that GDP affects the dynamic features of Gα is contradictory to previous research [55]. Regardless of the presence of GDP, the Gα structure bound to β_2_AR would be likely to show almost identical inter-domain dynamics, which indicates that GDP does not only stabilize sub-domain interactions but is consequentially released from Gα after opening of the nucleotide-binding pocket, which exposes its solvent-accessible surface. Regardless of whether β_2_AR binding event triggers the release of GDP directly or indirectly, it is unlikely that GDP causes large structural or dynamical changes in Gα.

Our ENI simulations also provided a possible pathway from the closed R-Gαβγ(GDP) complex to the fully opened R-Gαβγ(0) complex. In this process, the nucleotide-binding pocket is continuously and linearly exposed to the GDP-solvent area, increasing the possibility of GDP release. In addition, only the linker region between the two sub-domains of Gα changes in conformation, similar to a hinge point in the middle of two rigid bodies. Based on this result, we predict that the G protein signal cycle can potentially be regulated or inhibited by rendering this region flexible (S8 Fig).

GDP/GTP exchange was also studied by mean squared fluctuation calculations. Despite the small size of the GTP molecule, additional contacts between Gα and GTP could contribute to closing of the nucleotide-binding pocket and simultaneously dissociating of β_2_AR and Gβγ because of the high flexibility at the receptor- and Gβγ-binding interfaces of Gα.

As the last step in the G protein signaling cycle, GTP hydrolysis and Gβγ binding to Gα are thought to contribute to subsequent β_2_AR binding by decreasing the mobility of the N and C termini.

In conclusion, by studying the static and dynamic characteristics of the Gα structure, we gained novel structural insights into the mechanisms of G protein signaling. Even though some sequential events, such as the structural consequences of GTP/GDP binding to R-Gαβγ(0), are still under debate, we described for the first time the entire G protein signaling cycle (see S1 Video) with our ENM-based simulation model.

## Supporting Information

S1 Fig Process of making an engineered Gαβγ(GDP) model. (A) Comparison of the structures of Gα proteins from the Gs (1azt.pdb) and Gi (1gia.pdb) class. Despite the remarkable structural differences at loops region, the rmsd value between these two structures is very low, about 1.56 Å. (B) Comparison of the structures of active (1gia.pdb) and inactive (1gp2.pdb) Gαi. Switch 2 and linker 2 regions, which are Gβ and nucleotide binding regions, respectively, show marked topological differences except for the loop regions. (C) The sequence and topology of the engineered Gαβγ(GDP) model. The engineered model was constructed by combining residues from Gαs (residues 35–102 and 246–385) and in Gαi (residues 180–222, switch 2 and linker 2 regions).
(TIF)

S2 Fig Concept underlying the engineered R-Gαβγ(GDP) model. Gα in the engineered R-Gαβγ(GDP) model was constructed by combining the GαsRas domain from R-Gαβγ(0) and the GαsAH domain from engineered Gαβγ(GDP), respectively.
(TIF)

S3 Fig Schematic of the traditional ENM (*left*) and MWCENM (*right*). In the traditional ENM, the representative alpha carbons are colored in orange and their interactions within the cutoff distance of 12Å are shown as blue solid lines. In MWCENM, each representative atom is colored according to the types of amino acid. Chemical interactions are depicted by various types of lines. Black, blue, green solid and blue dashed lines represent backbone, ionic bonds, hydrogen bonds and van der Waals interactions within a cutoff distance of 8 Å, respectively.
(TIF)

S4 Fig Coarse-grained model of the nucleotide (GTP). Each representative atom defined in a dotted circle is weighted by the total mass of its surrounding atoms (shown in the same color) and connected to each other by covalent bonds. The coarse-grained model for GDP was exactly the same as that of GTP without the five atoms colored in blue.
(TIF)

S5 Fig B-factor correlation comparison of Gα in R-Gαβγ(0) [3sn6.pdb] between the traditional ENM (*white bar*) and MWCENM (*black bar*). Using various cutoff values from 8 to 16 Å, the traditional ENM showed cutoff dependency and relatively low b-factor correlation values under the 0.4, whereas the MWCENM had much more robust b-factor correlations, regardless of the distance cutoff values. The lowest correlation value with the cutoff of 8 Å in the traditional ENM was caused by an underconstrained system with sparse spring connections.
(TIF)

S6 Fig Effect of GDP on the dynamic behaviors of Gα. (A) Comparison of mean squared fluctuation values according to the presence or absence of GDP at R-Gαβγ and Gαβγ. In both structures, the correlation coefficients for Gαβγ (0.99) and R-Gαβγ (0.99) clearly indicated that GDP has little effect on the B-factor. (B) Comparison of NMA results of target structures based on cumulative square overlap (CSO) distributions over the first ten lowest modes. The CSO values of R-Gαβγ was almost 0.80, whereas that of Gαβγ was only about 0.60, regardless of the presence of GDP. Additionally, the opening motion of Gα occured at the 4th and the 5th modes only from R-Gαβγ(GDP) and R-Gαβγ, respectively; not from Gαβγ structures.
(TIF)

S7 Fig Virtual torsion angle change of the Gα structure from R-Gαβγ(GDP) to R-Gαβγ(0). The two peaks represent the residues for which the torsion angles varied significantly. Two linker parts presented in yellow in the Gα structure correspond to these regions. The dotted line, which represents the first linker but which is missing in our structures, is drawn on the basis of the existing structure of Gα (1gp2.pdb)
(TIF)

S8 Fig CSO comparison of R-Gαβγ(GDP) with a normal (blue) and a strong (red) constraint at linker regions. CSO over the first twenty lowest modes drops from 0.9 to 0.8 with a 100 times stronger spring constant under a two times wider range of cutoff around the linker residues.
(TIFF)

S1 Table Stiffness values in MWCENM. (PDF)

S2 Table Change in solvent-accessible surface area at the nucleotide pocket region and GDP in R-Gαβγ(GDP) through the open motion. Solvent-accessible surface area is calculated with PyMOL with 1 Å solvent radius.
(PDF)

S1 Video Animation showing the hypothesized conformational changes of the G protein throughout the G protein cycle. The GDP-bound heterotrimer (*gray*) binds to activated receptor (*blue*), and the nucleotide-binding pocket region consisting of β6-α5, the P-loop and switch 2 are perturbation, resulting in GDP (*purple*) release. Subsequently, the helical domain of Gα(GDP) opens away from the receptor-anchored Ras domain, which allows GDP release and GTP (*orange*) to enter. Reorientation of Gα caused by GTP binding leads to dissociation of the receptor and Gβγ (Gβ; *green* and Gγ; *cyan*). GTP hydrolysis leads to rebinding of Gβγ to Gα in order to restart the G protein signaling cycle.
(AVI)
