# Supplementary figures and images for: Computational Simulation of the Activation Cycle of Gα Subunit in the G Protein Cycle Using an Elastic Network Model
Source: PLoS One. 2016 Aug 2;11(8):e0159528. doi: 10.1371/journal.pone.0159528 (PMC4970668; doi:10.1371/journal.pone.0159528)

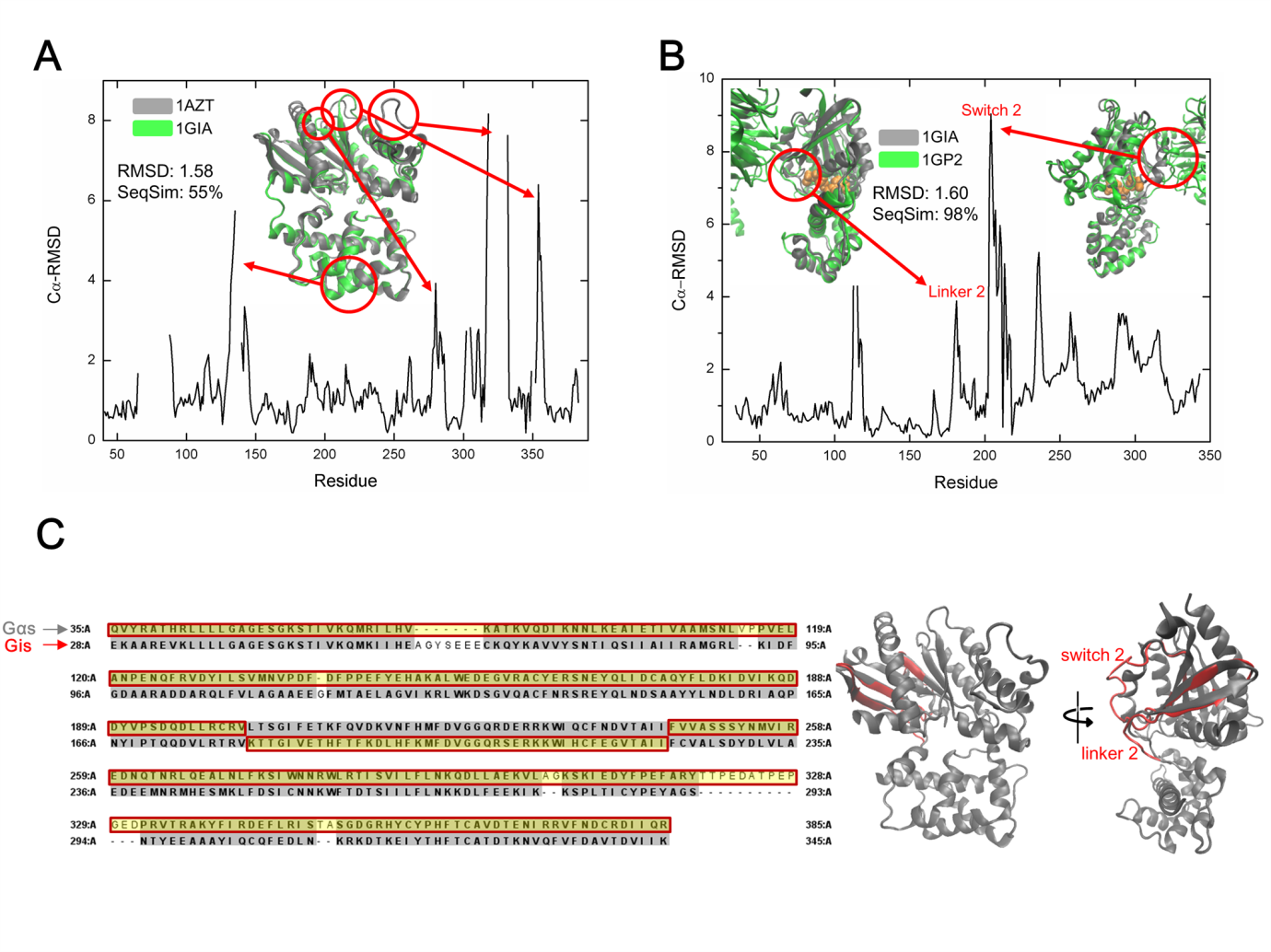

Supplement: S1 Fig — (A) Comparison of the structures of Gα proteins from the Gs (1azt.pdb) and Gi (1gia.pdb) class. Despite the remarkable structural differences at loops region, the rmsd value between these two structures is very low, about 1.56 Å. (B) Comparison of the structures of active (1gia.pdb) and inactive (1gp2.pdb) Gαi. Switch 2 and linker 2 regions, which are Gβ and nucleotide binding regions, respectively, show marked topological differences except for the loop regions. (C) The sequence and topology of the engineered Gαβγ(GDP) model. The engineered model was constructed by combining residues from Gαs (residues 35–102 and 246–385) and in Gαi (residues 180–222, switch 2 and linker 2 regions). (TIF) [file pone.0159528.s001.tif]

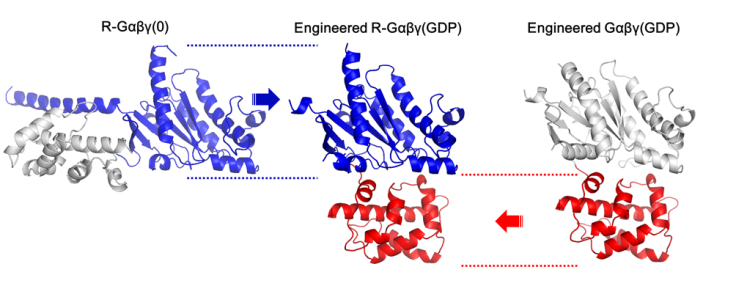

Supplement: S2 Fig — Gα in the engineered R-Gαβγ(GDP) model was constructed by combining the GαsRas domain from R-Gαβγ(0) and the GαsAH domain from engineered Gαβγ(GDP), respectively. (TIF) [file pone.0159528.s002.tif]

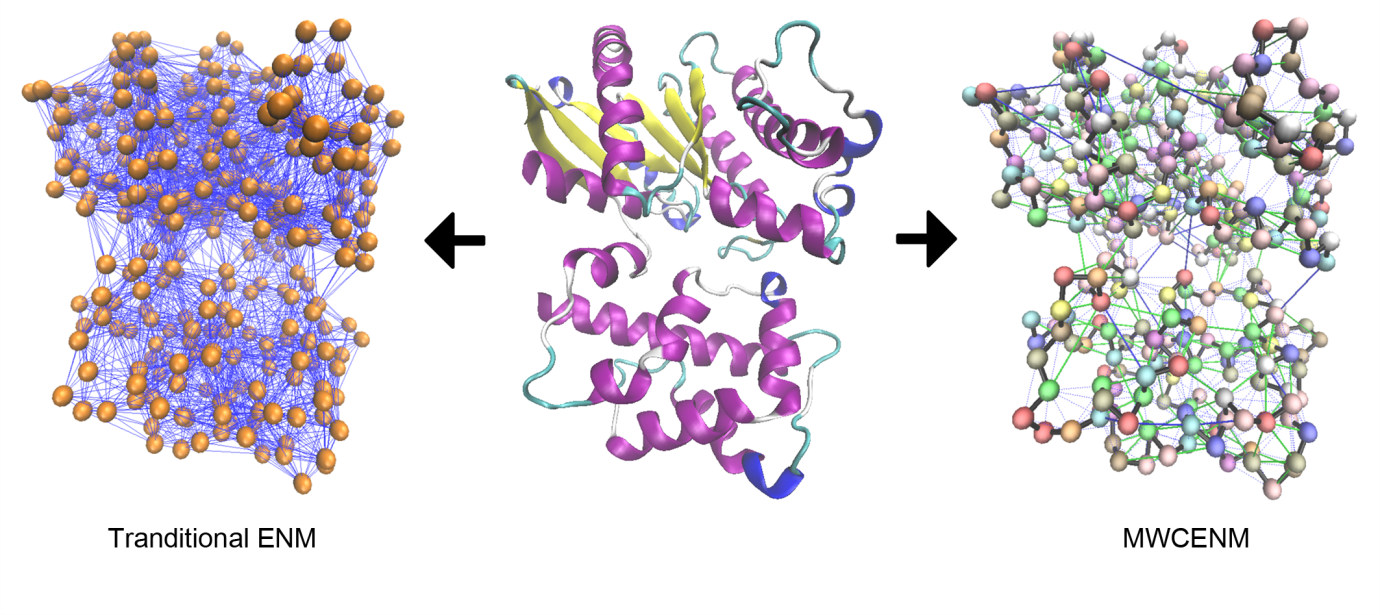

Supplement: S3 Fig — In the traditional ENM, the representative alpha carbons are colored in orange and their interactions within the cutoff distance of 12Å are shown as blue solid lines. In MWCENM, each representative atom is colored according to the types of amino acid. Chemical interactions are depicted by various types of lines. Black, blue, green solid and blue dashed lines represent backbone, ionic bonds, hydrogen bonds and van der Waals interactions within a cutoff distance of 8 Å, respectively. (TIF) [file pone.0159528.s003.tif]

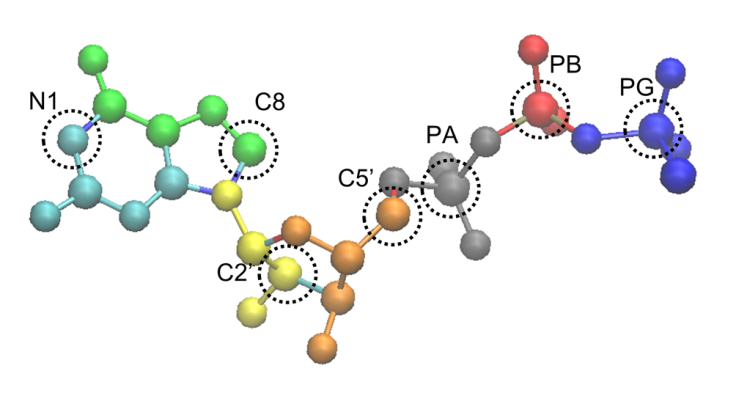

Supplement: S4 Fig — Each representative atom defined in a dotted circle is weighted by the total mass of its surrounding atoms (shown in the same color) and connected to each other by covalent bonds. The coarse-grained model for GDP was exactly the same as that of GTP without the five atoms colored in blue. (TIF) [file pone.0159528.s004.tif]

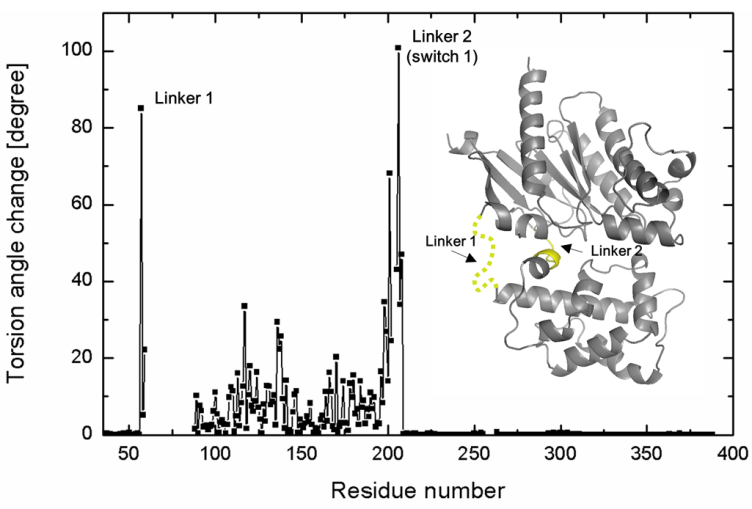

Supplement: S7 Fig — The two peaks represent the residues for which the torsion angles varied significantly. Two linker parts presented in yellow in the Gα structure correspond to these regions. The dotted line, which represents the first linker but which is missing in our structures, is drawn on the basis of the existing structure of Gα (1gp2.pdb) (TIF) [file pone.0159528.s007.tif]

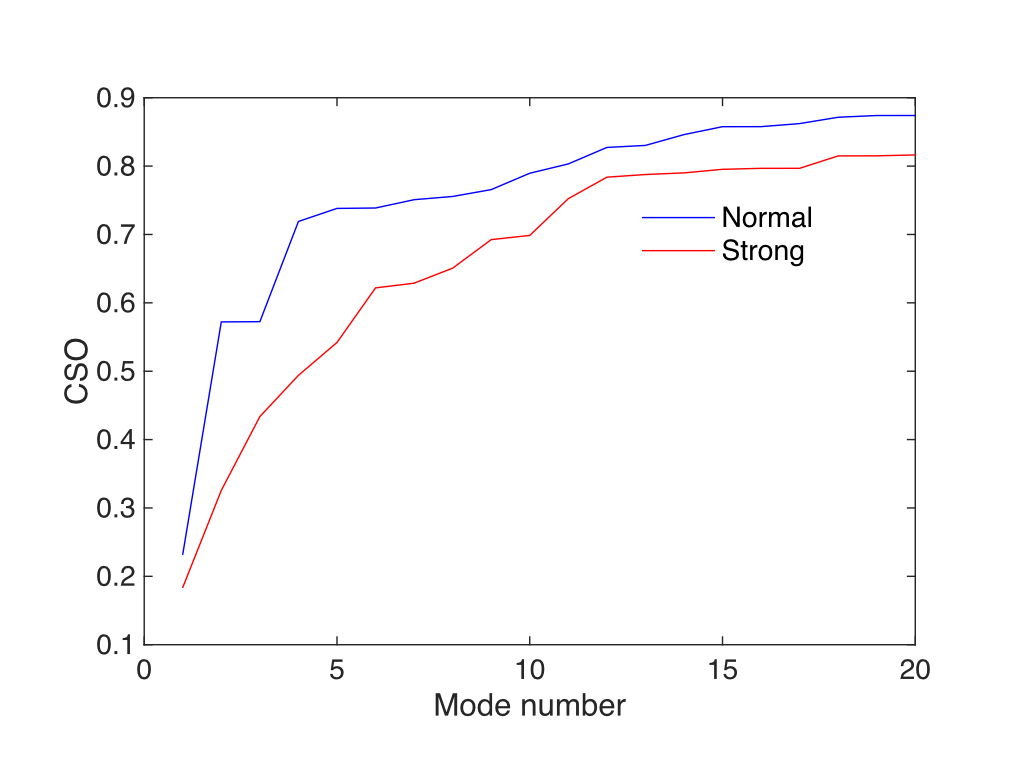

Supplement: S8 Fig — CSO over the first twenty lowest modes drops from 0.9 to 0.8 with a 100 times stronger spring constant under a two times wider range of cutoff around the linker residues. (TIFF) [file pone.0159528.s008.tiff]
