# Supplementary material for: Computational Simulation of the Activation Cycle of Gα Subunit in the G Protein Cycle Using an Elastic Network Model
Source: PLoS One. 2016 Aug 2;11(8):e0159528. doi: 10.1371/journal.pone.0159528 (PMC4970668; doi:10.1371/journal.pone.0159528)
Supplement: S1 Table — (PDF) [file pone.0159528.s009.pdf]

S1 Table

Stiffness values in MWCENM

Connection type	Stiffness ratio	Cutoff condition
Backbone (covalent)	100	Residue number (between ith and [i+1]th)
Backbone (non-bonded)	1	Residue number (between ith and [i+2/i+3]th)
Disulfide bond	100	PDB information
Hydrogen bond	10	HBPLUS [*]
Salt-bridge	10	Distance between charged residues < 4Å
Van der Waals force[†]	1	Non-bonded distance < 4Å
	$(4/d_{i,j})^8$	4Å $\leq$ Nonbonded distance, $d_{i,j}$ < 8Å

[*]HBPLUS is a program used to calculate all possible hydrogen bonds within a protein.

[†]The stiffness ratio for the second range of Van der Waals force interaction is only fitted by the attractive term of the Lennard-Jones potential.
