# Supplementary material for: Computational Simulation of the Activation Cycle of Gα Subunit in the G Protein Cycle Using an Elastic Network Model
Source: PLoS One. 2016 Aug 2;11(8):e0159528. doi: 10.1371/journal.pone.0159528 (PMC4970668; doi:10.1371/journal.pone.0159528)
Supplement: S2 Table — Solvent-accessible surface area is calculated with PyMOL with 1 Å solvent radius. (PDF) [file pone.0159528.s010.pdf]

S2 Table

Change of solvent-accessible surface area at nucleotide pocket region and GDP in R-Gαβγ(GDP) through the open motion

Region	β6-α5	P-loop	Switch2	GDP	Total
residue range	365-369	49-54	223-228	413	n/a
Initial structure ($Å^2$)	293.0	379.0	289.6	322.5	1284.1
NM structure ($Å^2$)	305.1	381.8	294.4	328.2	1309.5

Solvent-accessible surface area is calculated with PyMOL with 1 Å solvent radius.
